# A mathematical model to estimate cholesterylester transfer protein (CETP) triglycerides flux in human plasma

**DOI:** 10.1186/s12918-019-0679-x

**Published:** 2019-01-22

**Authors:** Martin Jansen, Gerhard Puetz, Michael M. Hoffmann, Karl Winkler

**Affiliations:** 1 Institute of Clinical Chemistry and Laboratory Medicine, Medical Centre - University of Freiburg, Freiburg im Breisgau, Germany; 2 Faculty of Medicine, University of Freiburg, Freiburg im Breisgau, Germany

**Keywords:** Enzymology/enzyme mechanism, Lipid transfer proteins, Lipoproteins/kinetics, Lipid transport, Lipoprotein metabolism, Mathematical model

## Abstract

**Background:**

Cholesterylester transfer protein (CETP) modulates the composition of various lipoproteins associated with cardiovascular disease. Despite its central role in lipoprotein metabolism, its mode of action is still not fully understood. Here we present a simple way to estimate CETP-mediated lipid fluxes between different lipoprotein fractions.

**Results:**

The model derived adequately describes the observed findings, especially regarding low- and high dense lipoproteins (LDL and HDL), delivering correlation coefficients of R^2^ = 0.567 (*p* < 0.001) and R^2^ = 0.466 (*p* < 0.001), respectively. These estimated fluxes correlate best among all other measured concentrations and ‘lipid per lipoprotein’ ratios to the observed fluxes.

**Conclusion:**

Our model approach is independent of CETP-action’s exact mechanistic mode. It is simple and easy to apply, and may be a useful tool in revealing CETP’s ambiguous role in lipid metabolism. The model mirrors a diffusion-like exchange of triglycerides between lipoproteins. Cholesteryl ester and triglyceride concentrations measured in HDL, LDL and VLDL are sufficient to apply the model on a plasma sample.

**Electronic supplementary material:**

The online version of this article (10.1186/s12918-019-0679-x) contains supplementary material, which is available to authorized users.

## Background

During their retention in human plasma the lipid load of lipoproteins is altered by several enzymes and receptors. Important enzymes are Cholesterylester transfer protein (CETP), lipases, lecithin-cholesterol acyl transferase (LCAT), and phospholipid transferprotein (PLTP). CETP mediates the transport of lipids, namely phospholipids (PLs), triglycerides (TG) and cholesteryl esters (CE) from one lipoprotein to another. TG and CE are strictly hydrophobic, are confined to the lipoprotein core, and cannot leave a lipoprotein by diffusion via the aqueous phase. However, the dynamics of CETP-mediated TG and CE fluxes resemble the dynamics of non-mediated diffusion [1]. Concentration gradients lead to a net flux of TG from TG-rich lipoproteins, namely very low density lipoproteins (VLDL) and intermediate density lipoproteins (IDL) to the more CE-rich lipoproteins low density lipoproteins (LDL) and high density lipoproteins (HDL). Correspondingly, CE is transported from LDL/HDL to VLDL/IDL. Consequently, in a drawn blood sample in vitro*,* the CE to TG ratio in all lipoprotein cores will ultimately reach equilibrium after within a certain period [2]. The CETP-mediated flux of CE from HDL to VLDL contributes to reverse cholesterol transport [3]. Although CETP is a key player in human lipoprotein metabolism, its impact on atherosclerosis and hypertension is not well understood. CETP inhibition has been a pharmaceutical approach to increase HDL cholesterol while decreasing LDL cholesterol and expecting a reduced risk of cardiovascular diseases. However, the clinical results of CETP-inhibition have been far from promising and deeper understanding of CETP action and the CETP-mediated dynamics between lipoproteins is urgently needed [4].

The exact molecular mechanism of lipid exchanges among lipoproteins via CETP has not been clarified. Two hypothesis are being debated: a) the ternary complex [5, 6], or b) a shuttle model [7].

In the ternary complex model, CETP binds to a lipoprotein followed by binding to another lipoprotein to a different side. In this situation CETP, forms a hydrophobic tunnel, enabling the exchange of neutral lipids between both lipoproteins (channel mechanism).

In the shuttle model, CETP docks to a single lipoprotein to receive one or two hydrophobic lipids, separates, and docks to another lipoprotein.

There are several methods of measuring CETP activity and mass concentration [8, 9]. As Lagrost [10] described, measurements of CETP activity may be classified into 2 categories: First, by measuring the net mass transfer of CE between lipoprotein fractions like VLDL, LDL and HDL, and second by using radiolabeled CE and observing its distribution over time among HDL and Apolipoprotein B-100 (ApoB) containing lipoproteins. Transport rates depend on various factors like the CETP mass concentration, number and composition of lipoproteins in plasma.

Aim of this investigation is to present a mathematical model that is independent of detailed molecular mechanisms, establishes a causal relationship of CETP-mediated fluxes between lipoproteins, and finally is able to estimate them.

## Results

### Subjects

Plasma samples in experiment 1 were divided into 4 subgroups: ‘normal’ (*n* = 43), ‘high TG’ (*n* = 18), ‘high LDL’ (*n* = 8) and ‘low HDL’ (*n* = 22) according to the methods described. Table 1 summarises the characteristics of all subjects.

**Table 1:** Baseline characteristics experiment 1 and 2

			CE					TG				
		Gender (M/W)	Serum	VLDL	IDL	LDL	HDL	Serum	VLDL	IDL	LDL	HDL
Experiment 1	• normal	28/15	3.74 (3.3,4.1)	0.21 (0.1,0.3)	0.11 (0.1,0.2)	1.90 (1.7,2.2)	1.22 (1.0,1.4)	0.85 (0.7,1.1)	0.50 (0.3,0.8)	0.06 (0.0,0.1)	0.15 (0.1,0.2)	0.09 (0.1,0.1)
	• high TG	13/5	5.00 (3.9,6.0)	0.61 (0.5,0.8)	0.19 (0.2,0.4)	2.76 (2.1,3.5)	0.97 (0.8,1.1)	2.17 (1.8,2.6)	1.68 (1.4,2.3)	0.09 (0.1,0.1)	0.25 (0.2,0.4)	0.11 (0.1,0.1)
	• high LDL	6/2	6.32 (5.6,7.4)	0.34 (0.3,0.5)	0.32 (0.2,0.5)	4.28 (3.5,4.7)	1.17 (0.9,1.3)	1.38 (1.2,1.5)	0.92 (0.8,1.1)	0.08 (0.1,0.1)	0.24 (0.2,0.2)	0.08 (0.1,0.1)
	• low HDL	13/9	3.17 (2.6,3.8)	0.24 (0.2,0.3)	0.10 (0.1,0.1)	1.77 (1.3,2.3)	0.85 (0.8,0.9)	1.18 (1.0,1.4)	0.81 (0.7,1.0)	0.05 (0.0,0.1)	0.15 (0.1,0.2)	0.10 (0.1,0.1)
	total	60/31	3.87 (3.2,4.8)	0.28 (0.2,0.5)	0.13 (0.1,0.2)	2.11 (1.7,2.7)	1.04 (0.9,1.3)	1.16 (0.9,1.6)	0.81 (0.5,1.2)	0.06 (0.0,0.1)	0.17 (0.1,0.2)	0.10 (0.1,0.1)
Experiment 2		6/5	3.38 (3.1,4.1)	0.20 (0.1,0.3)	0.09 (0.1,0.2)	1.77 (1.7,2.2)	1.12 (1.0,1.3)	0.79 (0.7,1.0)	0.49 (0.4,0.8)	0.05 (0.0,0.1)	0.12 (0.1,0.2)	0.08 (0.1,0.1)

Baseline characteristics experiment 1 and 2. CE and TG concentration are given as median (1st, 3rd quartile) in [mmol/L]. Experiment 1 is broken down into its 4 subgroups: ‘normal’, ‘High TG’, ‘High LDL’ and ‘Low HDL’ described in the method section

### Measurement imprecision

**Fig. 1:**
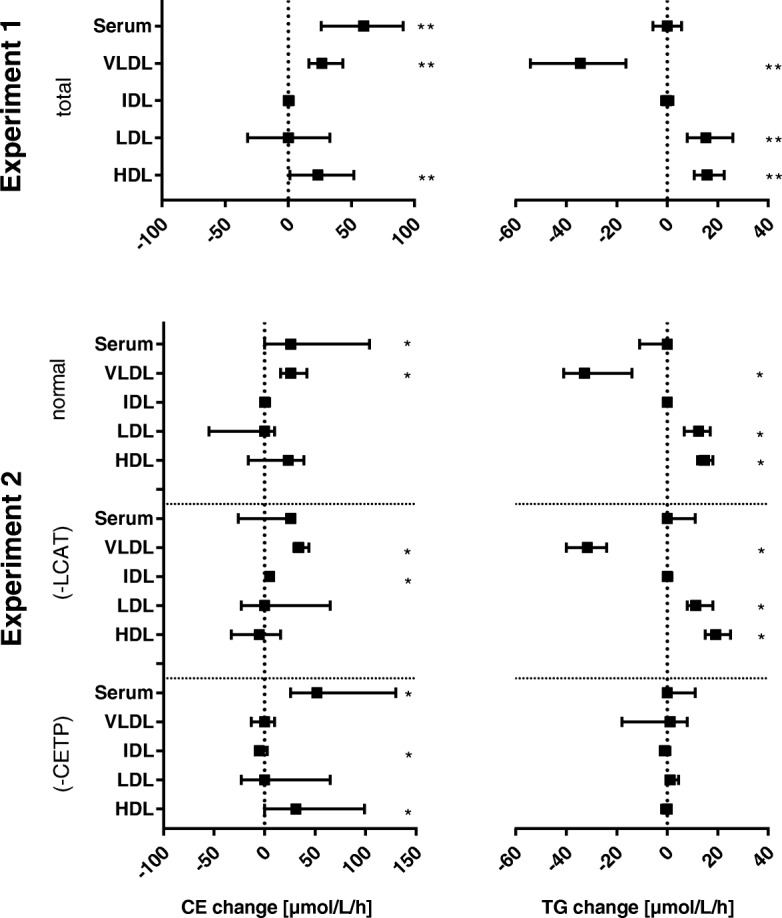
Changes in TG and CE due to plasma storage at 37 °C

The data used for model validation are based on small changes due to the 1 h storage at 37 °C in lipid mass of lipoprotein fractions (Fig. 1). Therefore, measurement imprecision was considered. It is less than 4% for all lipoprotein fractions studied. In most cases the expected changes after one hour at 37 °C are relatively small compared to baseline. Comparing the relative mean changes in TG mass to the corresponding coefficients of variation, the change in VLDL is relatively low, while it is high in LDL and HDL.

We used one hour as incubation duration, as longer durations may lead to non-linear behavior of lipoprotein associated reactions happening in vitro and hence to data, which is less comparable to the in vivo situation.

Changes in CE and TG between plasma stored at 4 °C and 37 °C for one hour, [μmol/L]. Median (1st, 3rd quartile) of differences between ‘37 °C value’-‘4 °C value’. Experiment 1: *n* = 91 samples of various metabolic states. Experiment 2: *n* = 11 plasma samples with no inhibition (normal), with inhibition of LCAT (-LCAT) and with inhibition of CETP (-CETP). Wilcoxon signed rank test * < 0.05, ** < 0.001.

Considering measurement imprecision, it is obvious that the data of TG-redistribution from a single plasma sample at 37 °C may be too noisy to yield acceptable inferences of real TG fluxes mediated by CETP. However, sufficiently large collectives (*n* ≥ 10) may enable us to test our model.

### CETP action

Figure 1 summarises changes in TG and CE concentrations in VLDL, IDL, LDL and HDL in experiments 1 and 2. The loss of TG in VLDL, as well as the increase in TG in LDL and HDL and the increase in CE in VLDL are significant in experiment 1. If CETP is inhibited (experiment 2), these changes are much smaller or even zero and lose their significance. If LCAT is inhibited the changes in TG in VLDL, LDL and HDL remain significant.

In experiment 1, there is a small but significant loss of 1% in VLDL ApoB (Wilcoxon: *p* = 0.001), but no significant corresponding changes in IDL or LDL. We assume that this ApoB is recovered in IDL or LDL but due to measurement imprecision we cannot statistically capture this fact.

### Correlations

**Fig. 2:**
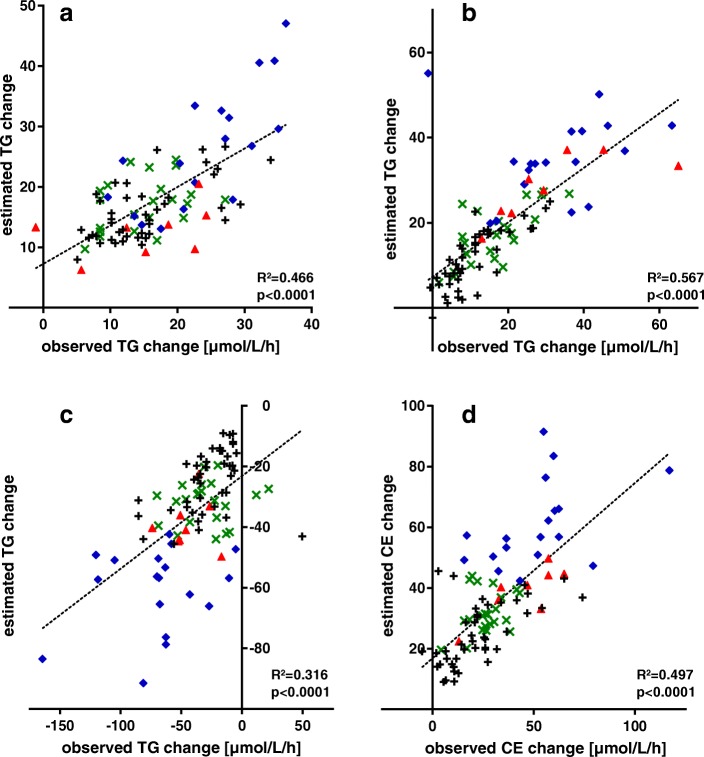
TG change in HDL (**a**), LDL (**b**) and VLDL (**c**) and CE change in VLDL (**d**) after 1 h plasma storage at 37 °C (observed vs modelled)

Figure 2 illustrates our model’s capability to estimate TG net flux via CETP in LDL, HDL and VLDL, as well as CE net flux in VLDL (assuming an equimolar exchange of TG and CE): Especially for HDL and LDL, the estimation correlates quite well to the measured changes. For VLDL, ΔCE correlates better than the corresponding ΔTG.

*N* = 91, subdivided into 4 metabolic states: *n* = 43 ‘normal’ (plus), *n* = 8 ‘high LDL’ (red triangle), *n* = 18 ‘high TG’ (blue diamond) and *n* = 22 ‘low HDL’ (green cross). Observed change: in TG (or CE) [μmol/L/h] in plasma stored at 37 °C. A: TG change HDL, B: TG change LDL, C: TG change VLDL, D: CE change VLDL (assuming equimolar exchange). Pearson’s correlation coefficient *R*^*2*^*.*

Table 2 lists the correlations between the measured fluxes of TG (and CE) to VLDL, IDL, LDL and HDL to the estimated fluxes. Additionally, the best correlation out of all our other measured parameters (including lipid and apolipoprotein masses, and the resulting lipid per apolipoprotein ratios) are listed. For HDL and LDL, model predictions are the best correlations among all tested correlations.

**Table 2:** Comparison between our model and other linear models based on observed data

	Lipoprotein fraction	R^2^ to model value	p	Next best correlation	R^2^ to other observed data	p
ΔTG	VLDL	0.316	6.8E^− 9^	PL in VLDL	0.312	8.7E^−9^
	IDL	0.188	1.7E^−5^	FC in VLDL	0.186	1.9E^−5^
	LDL	0.567	7.6E^−18^	ApoB in LDL-III	0.498	5.7E^−15^
	LDL-I	0.306	1.4E^−8^	Total CE	0.281	6.7E^− 8^
	LDL-II	0.759	2.8E^− 29^	ApoB in LDL-II	0.626	1.1E^−20^
	LDL-III	0.885	1.7E^−43^	ApoB in LDL-III	0.761	2.0E^−29^
	HDL	0.466	8.7E^− 14^	TG in VLDL	0.377	9.7E^−11^
Δ CE	VLDL	0.497	6.4E^−15^	Total TG	0.509	2.1E^− 15^
Δ TG/ApoB	VLDL	0.006	0.476	CE/ApoB in VLDL	0.089	4.0E^−3^
	IDL	0.092	3.0E^−3^	TG in IDL	0.076	1.1E^−2^
	LDL	0.441	7.3E^−13^	Total TG	0.391	3.5E^−11^
	LDL-I	0.217	3.0E^−6^	Total TG	0.257	2.9E^−7^
	LDL-II	0.386	5.2E^−11^	Total TG	0.273	1.1E^−7^
	LDL-III	0.623	1.6E^−20^	Total TG	0.503	3.5E^−15^
Δ TG/ApoA1	HDL	0.477	3.4E^−14^	TG in VLDL	0.368	2.6E^−9^
Δ CE/ApoB	VLDL	0.186	2.0E^−5^	ApoB in VLDL	0.160	8.7E^−5^

Correlation between observed and modeled change in TG (or CE) mass and change in TG (or CE) per ApoB (or ApoA1) in lipoprotein fractions in experiment 1 (*n* = 91). The corresponding best correlation out of all measured concentrations and lipid per apolipoprotein are also listed. Δ: baseline – plasma stored for 1 h at 37 °C

Note that in experiment 1 considering ΔTG, there is no significant correlation between HDL and LDL (*R* = 0.174, *p* = 0.098), but rather between VLDL and LDL (*R* = − 0.248, *p* = 0.018) as well as between VLDL and HDL (*R* = − 0.478, *p* < 0.0001), respectively.

In the VLDL fraction, there is no significant difference between LCAT- and non-LCAT-inhibited plasma in ΔCE. This suggests that LCAT activity in VLDL in experiment 2 is negligible.

Assuming an equimolar exchange between TG and CE in VLDL and no significant LCAT action on VLDL, the correlation between ΔTG and ΔCE in VLDL appears to be relatively weak (*R* = − 0.160, *p* = 0.130).

In contrast to the three LDL subfractions, the TG flux in the three measured HDL subfractions resulted in a much poorer fit compared to total HDL. This is not surprising, as the observed data are corrupted by the significant redistribution of ApoA1 mass among those fractions during the 1 h storage at 37 °C [11].

### CETP_TG_ and r_cetp_

**Fig. 3:**
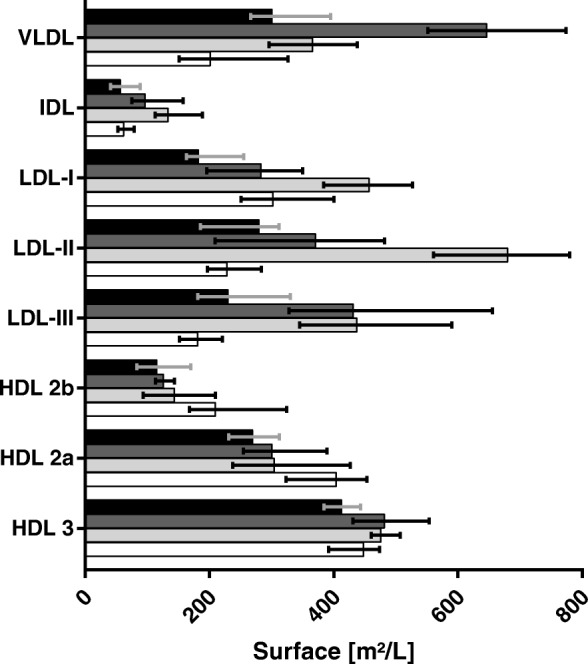
Calculated surface of lipoprotein fractions

Not surprisingly, correlations between the total calculated surface (as described in the method section) and corresponding PL concentration in HDL, IDL, LDL and VLDL are strong (*R* = 0.937, 0.994, 0.989 and 0.996, respectively). However, using calculated surfaces leads to slightly better results than using PL mass. Distribution of surfaces of lipoprotein (sub-)fractions is displayed in Fig. 3.

Calculated surfaces median (1st, 3rd quartile) of lipoprotein fractions in the different subgroups (‘normal’: white; ‘high LDL’: light grey; ‘high TG’: dark grey; ‘low HDL’: black).

**Fig. 4:**
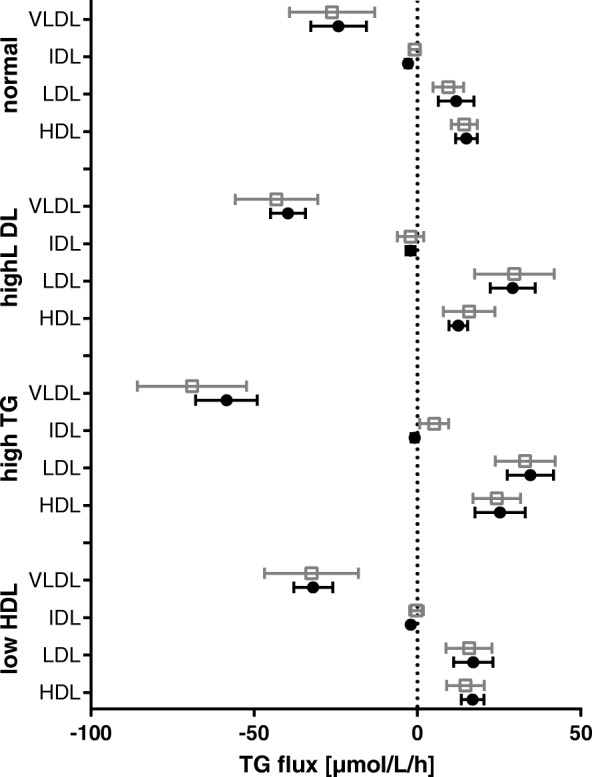
Predicted and observed TG fluxes

**Fig. 5:**
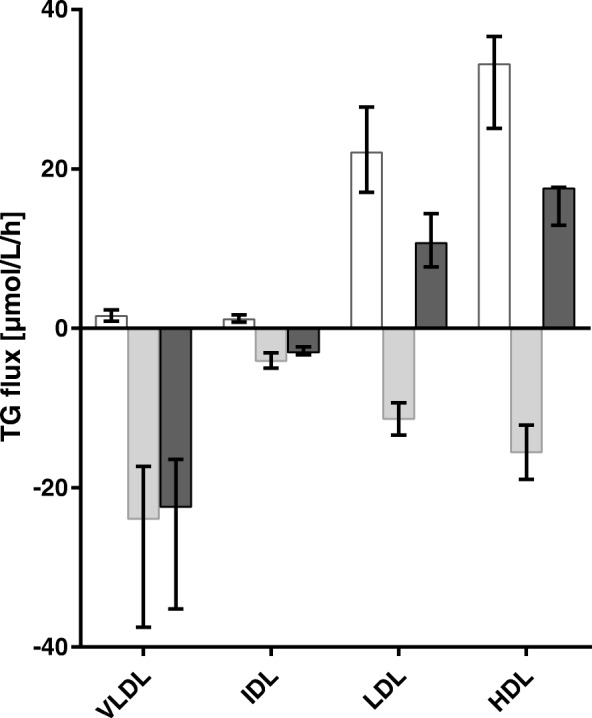
Predicted TG heteroexchanges

Figure 4 illustrates quartiles of the predicted and observed TG net fluxes for VLDL, IDL, LDL and HDL. Note that there is not only good correlation between the model and the observed data in each lipoprotein fraction, but the model is also capable of predicting the proportion of TG mass distributed among the lipoprotein fractions by CETP. The corresponding broken-down fluxes of TG into- and out of the fractions are displayed in Fig. 5.

Predicted TG net flux (black) and observed flux (white) of TG; Median (1st, 3rd quartile), 4 groups, *n* = 43/8/18/22, respectively.

Predicted median (1st, 3rd quartile) flux of TG entering via heteroexchange (white), leaving via heteroexchange (grey) and the resulting net flux (black) in ‘normal’ (*n* = 43).

**Fig. 6:**
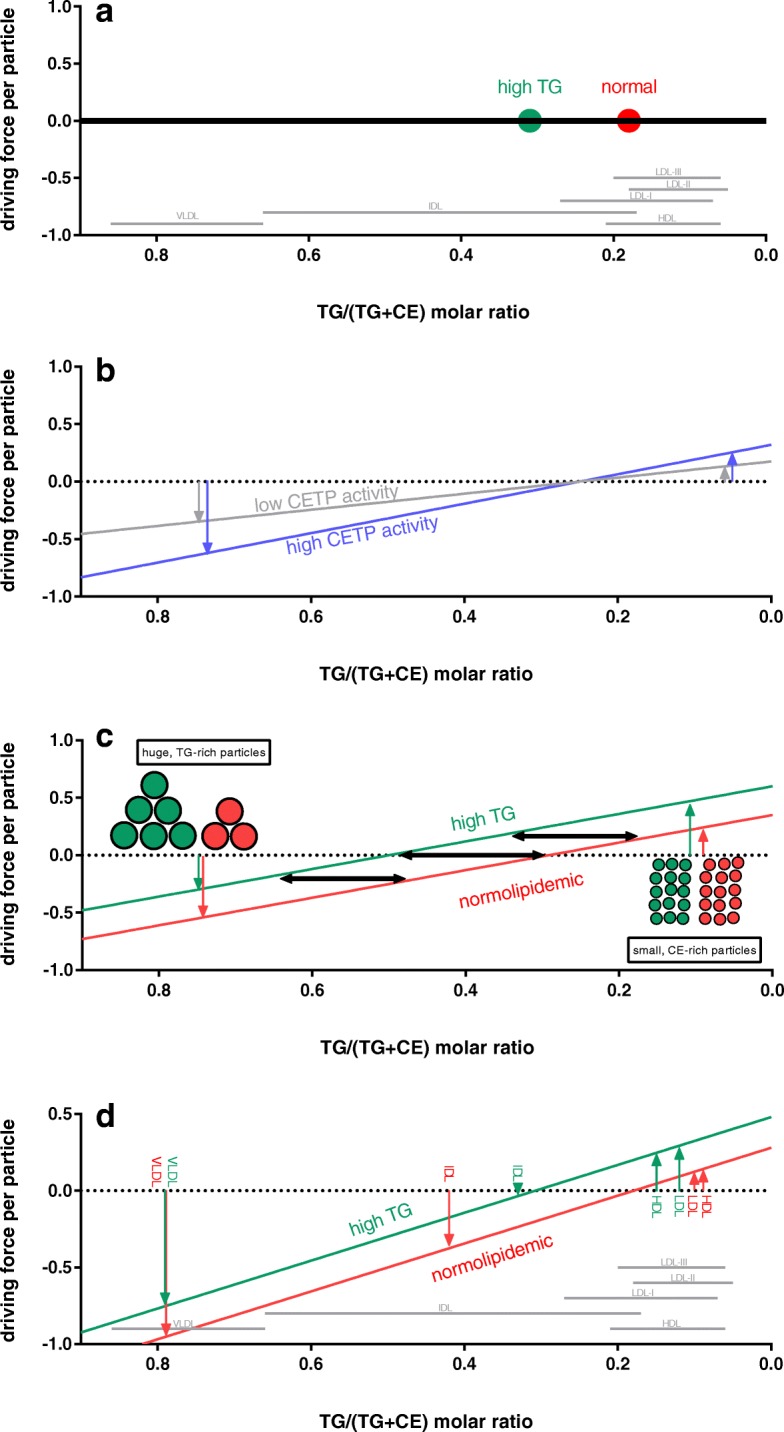
Isotransfer point (ITP) of CETP. **a** ITP: The green and the red point mark the ITP (the median of the v/v TG/(TG + CE) ratio of all lipoprotein particles weighted by their surface) for plasma of normal (*n* = 43) and hypertriglyceridemic (*n* = 18) persons, respectively. Grey lines mark the 95% confidence interval of the TG/(TG + CE) ratio, to which -based on n = 91 measurements of various metabolic states- the corresponding lipoprotein fraction can be allocated (LDL is subdivided into three subfractions). **b** CETP activity: The ITP is fixed. The influence of high or low CETP activity on the driving force of TG net change in lipoproteins per particle via CETP dependent on their TG/(TG + CE) is displayed. Particles on the right side of the ITP gain TG, particles on the left side loose TG. **c** Shift of the ITP: CETP activity is fixed. In a hyperlipidemic situation the ITP shifts to the left as (weighted by their surface) more TG-rich particles are present. In consequence the driving force per particle for CE-rich particles increases. **d** Concrete data: Arrows mark the driving forces per particle for VLDL, IDL, LDL and HDL in the normolipidemic (*n* = 43) and hyperlipidemic (*n* = 18) case

Figure 6 displays the *v*/v TG/(TG + CE) threshold at which –according to the model- there is no CETP mediated TG-net flux, a ratio that is here defined as “isotransfer point” (ITP). The here called ITP equals the parameter *CETP*_*TG*_*.* Considering all four metabolic situations investigated, the ITPs are always located between the corresponding means of the IDL and LDL fraction.

In experiment 1 the ITP shifts among the four subgroups: ‘high LDL’ 0.17 (0.16, 0.19), ‘normal’ 0.18 (0.15, 0.21), ‘low HDL’ 0.26 (0.21, 0.29), and ‘high TG’ 0.30 (0.26, 0.34). The correlation between CETP_TG_ and the ratio TG/(TG + CE) in plasma appears to be remarkably strong (*R* = 0.968, *p* = 3.7*E^− 55^).

The ITP equals a functional characteristic of lipoproteins in a defined metabolic state and shifts from LDL in normolipidemia to IDL in hyperlipidemia.

As described in the model the rate for CETP interacting with a lipoprotein in plasma per hour per lipoprotein surface in$$ \raisebox{1ex}{${m}^2$}\!\left/ \!\raisebox{-1ex}{$l$}\right. $$ is estimated as *r*_*cetp*_ *= 10*^− 5^*4.116.

## Discussion

Here we present a relative simple model for TG redistribution by CETP among lipoproteins in human plasma. Our model is based on the model of Potter et al. [1], which itself is based on the work of Morton [12, 13]. We relied on the data on TG composition of lipoproteins in plasma stored for one hour at 37 °C of *n* = 91 samples compared to baseline to calculate the CETP-mediated TG-redistribution among lipoproteins.

A slightly different version of the model presented here was already introduced in our group’s published article [11]. The main difference from that model is that in the present work, we are relaxing the assumption that exchanges are equimolar. In our earlier model, only the means of TG in LDL (*n* = 27, normolipidemic) were used for model calibration. However, in this study, we not only acquired more data from both healthy and pathologic lipoprotein profiles to test the model, we also expanded our model to include VLDL and HDL. Moreover, we do not only compare measured and estimated medians but also considered corresponding correlations.

### The model’s structure

Potter’s model describes an equimolar shuttle exchange of TG and CE by CETP between lipoproteins on the molecular level. Hence, on a formal basis, CETP is discriminated into a free and a lipoprotein-bound fraction. Correspondingly, lipoproteins are discriminated into free and CETP-bound. The model presented here simplifies and generalises this model. Our model is independent of the exact biochemical mode of CETP action (shuttle or ternary complex) - an obvious advantage, since the mode of action has still not been elucidated. Our generalised model is compatible with both the shuttle and the ternary complex models. Although differing on the molecular level, both approaches result in the same dynamics on the macroscopic level (by the law of large numbers).

As we point out later, our data do not support equimolar change in all fractions, thus we model the TG change only. However, at least in VLDL, our model seems to predict the CE flux to a strong degree. Furthermore, we are not modelling free and bound lipoproteins and CETP-molecules explicitly. The ‘CETP binding to lipoprotein’-reaction is disregarded, as the dynamics of those reactions are only relevant on a mechanistic level.

Note that there are several mathematical model approaches in the field of human lipid metabolism dealing with for example topics like oxidized lipoproteins [14], LDL endocytosis [15] or the cholesterol biosynthesis pathway [16].

### Comparison to other models

**Fig. 7:**
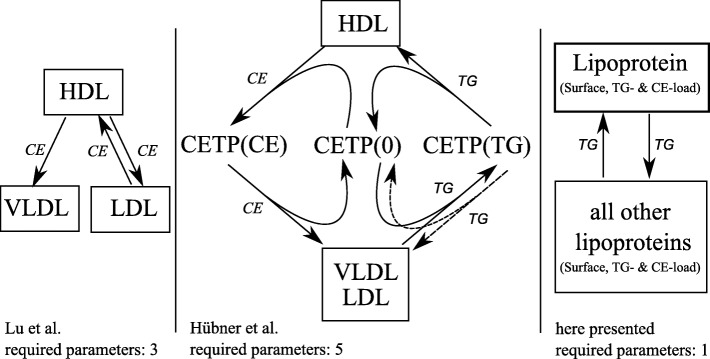
Comparison between different CETP models

There are other models besides Potter’s describing in vivo CETP dynamics. Figure 7 summarises two thereof. The model presented by Lu et al. [17] describes the flux of CE from HDL to VLDL as well as CE’s flux from LDL to HDL and vice-versa. In Lu’s model the fluxes are simple first-grade reactions${R}_i:C{E}_{donor}\overset{k_i}{\to }C{E}_{acceptor}$ (*i* = 1, 2, 3) with three different corresponding reaction rates. Applying our own data to both models, the observed ΔCE correlates much weaker to Lu’s predicted ΔCE in VLDL than to our predicted ΔCE: *R* = 0.315, *p* = 0.002 vs. *R* = 0.705, *p* = 6.4E^− 15^. As we are not assuming equimolar exchange and just model TG, we cannot compare Lu’s model directly to ours. However, considering the LCAT-inhibited data from experiment 2, we detected no correlation between the CE enrichment in HDL and CE mass in LDL (or VLDL).

Hübner’s model [18] considers TG and cholesterol (CH), which corresponds to FC + CE. A shuttle transport is modeled in which CETP is either free or TG- or CH-loaded. Let us replace CH by CE to better compare their model to ours. Here, five rates are used for five reactions (Fig. 7). Their model neglects CETP-mediated transport of TG out of HDL, and CE out of ApoB-containing particles. However, their model somehow makes use of the ratio of TG to (CE + TG) associated to CETP. Comparing the strength of correlation between Hübner’s model and our model, ours appears to be superior with regard to HDL-ΔTG, LDL-ΔTG, and slightly better for VLDL-ΔTG (*R* = 0.515, *R* = 0.563, *R* = 0.544 vs our model’s: *R* = 0.683, *R* = 0.753, *R* = 0.562, respectively).

Comparison between the CETP model of Lu et al. [17], Hübner et al. [18] and ours presented. Lu’s model uses first-order reactions; Hübner’s model uses 2 second-order and 3 first-order reactions. The model presented here assumes a steady state and uses one reaction rate for CETP. Due to CETP’s diffusion-like property, the amount of TG entering and leaving a lipoprotein depends on the surface and the TG and CE load of the lipoprotein and all other lipoproteins.

Comparing the Hübner and Lu models to ours presented here, note that ours is less complex, as it only requires one reaction rate instead of 5 or 3, respectively. Hence, unlike their models, which describe each flux from one fraction to another with their own reaction, our newly derived model represents a rather holistic model.

### Concept of isotransfer point (ITP)

Figure 6a displays the medians of the ITP in the ‘normal’ and the ‘high TG’ group. Intuitively speaking, the point is lower in the ‘normal’-group. Based on our model, the ITP ‘CETP_TG_’ is the mean of all fraction’s TG/(TG + CE) ratios weighted by the corresponding surface. As clarified in Fig. 6 the greater the distance between the TG/(TG + CE) ratio of a lipoprotein to the ITP, the higher is the net flux of TG into/out of this fraction (if the ratio is smaller/greater than the ITP). Thus we expect LDL and HDL to have a higher TG turnover in the ‘high TG’ situation.

The ITP may be changed due to lipid-lowering medication or during the postprandial state. This change may also exert a significant influence on HDL metabolism. The TG/(CE + TG) ratio in IDL in the ‘high TG’ group is surprisingly low. This might be caused by a prolonged retention time in plasma, which may be characteristic in the hypertriglyceridemic state. The clinical significance of the shift of the ITP from LDL to IDL in hyperlipidemia remains unclear. However, the atherogenity of certain lipoproteins (e.g. IDL) may be altered if its metabolic function (CETP-mediated TG transfer) changes.

### Data

Our in vitro data mirror the corresponding in vivo redistribution of lipids to a fairly good degree, as data from Liu et al. [19] as well as our group’s experiments have revealed evidence that the net change in TG in lipoprotein fractions by CETP action during plasma storage at 37 °C is linear for at least four hours.

Further data from experiment 2 suggest that CETP is the only factor responsible for TG redistribution among lipoprotein subfractions. CETP inhibition suggests that the esterification rate on non-HDL is very low or even non-existent. However, the data may be too noisy to make precise claims.

In contrast to other methods measuring CETP-activity, the data we used to estimate the CETP-mediated TG-change enables the estimation of TG in all fractions, namely VLDL, IDL, LDL and HDL, simultaneously. The data cover a broad physiological range of TG changes mediated by CETP, as individuals with healthy and various pathologic lipid profiles have been considered.

Very probably CETP activity differs among individuals. However, as the first step, our model estimates the CETP-mediated relative redistribution of TG among the lipoproteins, which is independent of the CETP reaction rate, given only the TG, CE and ApoB (or ApoA1) concentration of VLDL, IDL, LDL and HDL. Based on this estimation, absolute fluxes may be inferred assuming a fixed reaction rate in a second step, if no additional information is given.

### Model fit

Despite the noise of TG change data in lipoprotein fractions, the predicted TG fluxes correlate well with the observed corresponding changes (Fig. 2). While HDL and LDL fit well, VLDL and especially IDL appear more problematic. This could be mainly caused by relatively greater measurement imprecision, but also by a shift of ApoB from IDL to VLDL or vice-versa during the one hour storage at 37 °C – as our group already observed [11]. Considering the R^2^ values between modelled and measured TG changes in VLDL, LDL and HDL (Fig. 2), mind that on the one hand our rather imprecise method of measurement may strongly lower the ‘true’ R^2^ value and on the other hand that our prediction is superior compared to the other models mentioned above, as well as to possible linear correlations mentioned in the results part (Table 2).

### Equimolarity

Assuming an equimolar lipid-exchange it is expected that the amount of TG entering/leaving a lipoprotein fraction equals the amount of CE leaving/entering it if LCAT inhibition is given. Considering ΔCE and ΔTG in experiment 2 (Fig. 1) we noted a significant increase in the TG-median of 11.3 μmol/L and 19.2 μmol/L in LDL and HDL under LCAT inhibition. Surprisingly no corresponding loss of CE (0 and − 5.2 μmol/L, respectively) was observed. However, due to the small sample size and measurement imprecision, we cannot make a solid statement on this point.

Taken together, we identified some indicators suggesting that the CETP exchange may not necessarily be equimolar. However, due to experiment 2’s low case numbers, our data do not suffice to clarify this issue.

### Lipid transfer inhibitor protein (LTIP)

There is strong evidence of a protein inhibiting CETP, namely lipid transfer inhibitor protein (LTIP) or Apolipoprotein F, which mainly interacts with LDL particles [20]. We investigated whether the resulting lower availability of LDL would have a positive effect on our model’s fit by reducing *s(P*_*LDL*_*)*, the surface of LDL. Potter uses a factor of 0.73 for LDL inhibition by LTIP. Such a reduction in our case leads in fact to a better correlation between predicted and observed changes in LDL and especially HDL: If *s(P*_*LDL*_)*0.4 is used instead of *s(P*_*LDL*_*)*, the correlation is optimised. If *s(P*_*LDL*_*)* is reduced, the correlation coefficients *R* in HDL and LDL increase slightly, while the corresponding coefficient in VLDL decreases simultaneously. Further the estimated and observed proportion of TG enrichment between LDL and HDL tends to impair as *s(P*_*LDL*_*)* is reduced. A possible inhibitory effect of ApoF is thus not implemented in our model.

### CETP mass

Estimating TG fluxes in experiment 1, we assumed all *r*_*cetp*_ to be equal. This of course did not hold true. The parameter *r*_*cetp*_ rather depends on CETP’s mass concentration and the ability of the particular CETP-phenotype to mediate lipid exchange.

In the literature, CETP’s plasma concentration does not differ that much depending on gender or pathologies [8, 21–25]. We are thus assuming that the mass is equal in experiment 1’s different subgroups. However, our model may not account for changes in *r*_*cetp*_ due to genetic variants of CETP.

### Properties of CETP exchange

Considering our model and applying it to several lipid-profiles, two issues deserve attention: First, the driving force for CETP exchange is the mass and composition of triglyceride-rich lipoproteins such as VLDL and chylomicrons, as they contain the most TG in plasma. Figure 4 illustrates this fact, as in the ‘high TG’ case, LDL and HDL both revealed the highest increase in TG mass. This issue might be an important factor, when assessing TG influence on lipid metabolism (for example the postprandial state).

Second, TG from VLDL is distributed to LDL and HDL to a similar degree in all four metabolic states investigated (Fig. 4). However, depending on their surface and lipid composition, the proportion of TG entering HDL compared to TG entering LDL may differ strongly, as both lipoprotein-species compete against each other (consider for example the ‘high LDL’ case in Fig. 4).

Let us assume that TG’s high flux to LDL or HDL is atherogenic, as (in combination with hepatic lipase) it can lead to small, dense LDL and small HDL. Following the model of Lu et al. [17], lipolysis by hepatic lipase is the main pathway causing HDL particles to shrink. Small HDL are more likely to exit the plasma. Hence, via CETP hypertriglyceridemic plasma may lead to an atherogenic lipoprotein phenotype characterized by low HDL particle concentrations and small HDL and LDL particles. Our model might thus help to quantify the causal relationship between hypertriglyceridemia and those atherogenic characteristics. Furthermore, following our model’s dynamics, a high HDL particle concentration may prevent small dense LDL particles from forming by reducing the net flux of TG to LDL in such a hypertriglyceridemic state. However, the focus of this paper making is not claims about HDL functionality by relying on its lipid-composition.

Note that only 43 normolipidemic persons out of experiment 1 were used for model calibration. Hence, the results for ‘high TG’, high LDL’ and ‘low HDL’ presented in Fig. 4 are extrapolated and demonstrate our model’s ability to predict CETP redistribution of TG based on lipid and apolipoprotein concentrations only.

When studying lipoprotein metabolism, the concepts considered are usually delipidation of ApoB containing lipoproteins and the reversed cholesterol transport mediated by HDL. However, both metabolic pathways are intimately connected by the CETP-mediated flux of TG and CE. This paper is intended to give a new perspective on lipoprotein metabolism concerning CETP-mediated fluxes of TG and CE.

## Conclusion/outlook

Our newly devised model may be employed to estimate CETP-flux data among different lipoprotein fractions using only CE, TG, ApoA1, and ApoB concentrations in lipoprotein fractions. Our model’s structure relies on the known biochemical characteristics of CETP, but is independent of its molecular transport mechanism. The means we have presented to estimate CETP action may help to deepen our understanding of CETP’s role in several physiological and pathological scenarios. For example, lipoprotein metabolism in the postprandial state remains difficult to access, as there is strong TG redistribution from chylomicrons to other lipoproteins. Hence, delayed catabolism of chylomicrons may exert strong effects on HDL and LDL metabolism. Thus, applying our approach to address CETP in such conditions it may now be possible to also make more accurate predictions even in the postprandial state.

## Methods

### Subjects & experimental procedure

For experiment 1 we used *n* = 91 plasma samples from individuals with a broad range of hyper-, normo- and hypolipidemic states. Plasma samples are broken down into 4 disjoint subgroups:

- ‘normal’: TG < 1.65 mmol/L, LDL-Cholesterol< 4.03 mmol/L, HDL-Cholesterol> 1.04 mmol/L for males and > 1.17 mmol/L for women;
- ‘high TG’: Total TG ≥ 1.65 mmol/L;
- ‘high LDL’: TG < 1.65 mmol/L, LDL-Cholesterol≥4.03 mmol/L;
- ‘low HDL’: TG < 1.65 mmol/L, LDL-Cholesterol< 4.03 mmol/L, HDL-Chol≤1.04 mmol/L for males and ≤ 1.17 mmol/L for women

For experiment 2 we used plasma samples of *n* = 11 volunteers (n = 9 normal, *n* = 2 non-normal). The limits of subgroup ‘normal’ are based on guidelines from the German medical association ‘Deutsche Gesellschaft zur Bekämpfung von Fettstoffwechselstörungen und ihren Folgeerkrankungen DGFF (Lipid-Liga) e. V.’ (https://www.lipid-liga.de/fuer-aertze/empfehlungen). We followed a method described by Jansen and colleagues [11]. In short, EDTA-plasma was separated into two samples for both experiments. While the first was stored at 4 °C immediately, the second was exposed to 37 °C for one hour (water bath) and then stored at 4 °C as well. Subsequently, lipoprotein fractions were isolated via ultracentrifugation as described before [26]. Lipoproteins were separated into VLDL, IDL, LDL, HDL and lipid deficient serum (LDS) and further into three LDL subfractions LDL-I, LDL-II and LDL-III and three HDL subfractions HDL2b, HDL2a and HDL3. TG, CE, free cholesterol (FC), PL as well as ApoB and Apolipoprotein A1 (ApoA1) were measured in plasma and each separated lipoprotein fraction. Further the ratios ‘lipid to ApoB’ and ‘lipid to ApoA1’ were calculated to estimate the lipoprotein composition. These values were measured with storage at 37 °C or 4 °C, the corresponding differences in lipid and apolipoprotein mass and lipoprotein composition were calculated as the differences of baseline (4 °C) minus the 1 h 37 °C value and denoted with Δ.In experiment 2, each plasma sample was additionally divided into 4 groups in which only CETP, only LCAT, or none of both enzymes were inhibited. CETP was inhibited via Torcetrapib (1 μM, resolved in dimethyl sulfoxide), LCAT was inhibited by sodium iodacetate (5 mM resolved in Tris buffer [27]).

The coefficient of variation (CV: SD/mean*100) was determined by taking 20 repeated measurements of lipoprotein fractions.

### The model

This work is based on the model by Potter et al. [1], which was modified by our group recently [11]. Briefly, metabolism of FC, CE and TG in LDL was modelled on the molecular level. In contrast to the recently published model, which assumed a one-to-one exchange of TG and CE, this restriction for equimolarity is now relaxed, as only TG are considered for flux calculations. If CETP mediates an exchange between two lipoproteins (ternary model) or between a CETP loaded with CE or TG (shuttle model), neither CE nor TG is preferred. Further, in line with Morton [12, 13] we assume that the probability of the association of CETP to a lipoprotein is proportional to its surface. To simplify our model, the lipoprotein core consists of only TG and CE.

Let us consider the situation in which a CETP interacts with a lipoprotein particle. We are assuming that two independent events will occur:

- Event 1: a TG molecule leaves the lipoprotein *or* no TG molecule leaves the lipoprotein
- Event 2: a TG molecule enters the lipoprotein *or* no TG molecule enters the lipoprotein

Given those 2 events, a CETP interaction can lead to 4 possible outcomes:

- A TG leaves the lipoprotein (heteroexchange)
- A TG enters the lipoprotein (heteroexchange)
- A TG enters *and* a TG leaves the lipoprotein (homoexchange)
- No TG enters or leaves the lipoprotein

Considering event 1, the probability that a TG molecule leaves the particle equals the particle’s $\frac{TG}{CE+ TG}$ *v*/v ratio and the complementary probability (no TG molecule leaves the particle) equals the particle’s $\frac{CE}{CE+ TG}$ *v*/v ratio.

Considering event 2, the probability that a TG molecule enters the particle equals a parameter called *CETP*_*TG*_ while *(1- CETP*_*TG*_*)* is the complementary probability (no TG molecule enters the particle).

The parameter *CETP*_*TG*_ is derived out of lipoprotein surfaces and TG and CE concentrations: *CETP*_*TG*_ mirrors the $\frac{TG}{CE+ TG}$ *v*/v ratio of all TG and CE available from all lipoprotein-particles (weighted by their surface) in plasma to CETP. Let *P*_*i*_ denote the *i*-th lipoprotein fraction (characterised by its mean TG and CE composition), where *i* ∈ *I* = {VLDL, IDL, LDL − I, LDL − II, LDL − III, HDL2b, HDL2a, HDL3}. For each fraction *P*_*i*_ respective its particle-surface *S(P*_*i*_*)*, its concentration of particles *k(P*_*i*_*)*, its $\frac{TG}{CE+ TG}$ v/v ratio *q(P*_*i*_*)* and its $\frac{CE}{CE+ TG}$ v/v ratio *q*^′^(*P*_*i*_) = 1 − *q*(*P*_*i*_) can be allocated. Consequently, the surface (corresponding to the given particle concentration) of all lipoproteins equals$\sum \limits_{i\in I}s\left({P}_i\right)k\left({P}_i\right)$. It holds for *CETP*_*TG*_, the $\frac{TG}{CE+ TG}$ v/v ratio of all lipoprotein-particles weighted by their surface:

$$ {CETP}_{TG}=\raisebox{1ex}{${\sum}_{i\in I}q\left({P}_i\right)s\left({P}_i\right)k\left({P}_i\right)$}\!\left/ \!\raisebox{-1ex}{${\sum}_{i\in I}s\left({P}_i\right)k\left({P}_i\right)$}\right. $$

The methods applied for calculating a particle’s surface and the concentration of particles in the HDL subfractions are described previously [11].

Taken together, given an interaction of CETP with a lipoprotein of fraction *P*_*i*_, the lipoprotein acquires a TG molecule in total, if no TG molecule leaves the particle and one TG molecule enters the particle. The corresponding joint probability is *q*^′^(*P*_*i*_) ∙ *CETP*_*TG*_. The probability of a TG molecule leaving the lipoprotein in total can be derived analogously as *q*(*P*_*i*_) ∙ (1 − *CETP*_*TG*_). These exchanges are so-called heteroexchanges, as a TG molecule is replaced by a non-defined number (due to the assumption of non-equimolary exchange) of CE molecules. The corresponding probability of a TG-homoexchange (a TG leaves and a TG enters the lipoprotein) is *q*(*P*_*i*_) ∙ *CETP*_*TG*_.

Consequently, the fluxes of TG to the quadruple [VLDL, IDL, LDL, HDL] are proportional to

$$ {r}_{cetp}{CETP}_{TG}\left[\begin{array}{c}k\left({P}_{VLDL}\right)s\left({P}_{VLDL}\right){q}^{\prime}\left({P}_{VLDL}\right)\\ {}\begin{array}{c}k\left({P}_{IDL}\right)s\left({P}_{IDL}\right){q}^{\prime}\left({P}_{IDL}\right)\\ {}k\left({P}_{LDL}\right)s\left({P}_{LDL}\right){q}^{\prime}\left({P}_{LDL}\right)\end{array}\\ {}k\left({P}_{HDL}\right)s\left({P}_{HDL}\right){q}^{\prime}\left({P}_{HDL}\right)\end{array}\right]\kern0.5em \left( term\ 1\right) $$

where *r*_*cetp*_ is the reaction rate of CETP. The corresponding fluxes of TG out of the quadruple [VLDL, IDL, LDL, HDL] are proportional to

$$ {r}_{cetp}\left(1-{CETP}_{TG}\right)\left[\begin{array}{c}k\left({P}_{VLDL}\right)s\left({P}_{VLDL}\right)q\left({P}_{VLDL}\right)\\ {}\begin{array}{c}k\left({P}_{IDL}\right)s\left({P}_{IDL}\right)q\left({P}_{IDL}\right)\\ {}k\left({P}_{LDL}\right)s\left({P}_{LDL}\right)q\left({P}_{LDL}\right)\end{array}\\ {}k\left({P}_{HDL}\right)s\left({P}_{HDL}\right)q\left({P}_{HDL}\right)\end{array}\right]\kern0.5em \left( term\ 2\right) $$

The net flux of TG is proportional to the sum of term 1 and term 2:

$$ {r}_{cetp}\left[\begin{array}{c}k\left({P}_{VLDL}\right)s\left({P}_{VLDL}\right)\left(q\left({P}_{VLDL}\right)-{CETP}_{TG}\right)\\ {}\begin{array}{c}k\left({P}_{IDL}\right)s\left({P}_{IDL}\right)\left(q\left({P}_{IDL}\right)-{CETP}_{TG}\right)\\ {}k\left({P}_{LDL}\right)s\left({P}_{LDL}\right)\left(q\left({P}_{LDL}\right)-{CETP}_{TG}\right)\end{array}\\ {}k\left({P}_{HDL}\right)s\left({P}_{HDL}\right)\left(q\left({P}_{HDL}\right)-{CETP}_{TG}\right)\end{array}\right] $$

With *k*, *s* and *q* calculated by measured data, only the reaction rate *r*_*cetp*_ needs to be inferred to calibrate our model. The proportion of TG mass entering and leaving the single fractions is already determined by the model’s structure (it depends on TG, CE and particle concentrations in the single fractions). We used the subgroup ‘normal’ (*n* = 43) to infer *r*_*cetp*_ by minimising the difference between measured and predicted TG net flux in VLDL, IDL, LDL and HDL (weighted by the corresponding surface). All other non-‘normal’ cases were used as a validation group.

The Wilcoxon signed rank test was performed to test for differences between the change in CE and TG due to plasma storage at 37 °C for one hour. The Pearson’s correlation coefficient *R* was used to describe relationships between variables.

Statistics were analysed with IBM SPSS version 21.0 (IBM SPSS Statistics, IBM Corporation, Chicago, IL), all other computations were done using Scilab 5.4.0 (Scilab enterprises, Le Chesnay). Graphs were depicted by GraphPad Prism Software V7.00.

## Additional file

Additional file 1: Lipoprotein data of all subjects. (CSV 35 kb)

## Abbreviations

ApoA1: Apolipoprotein A1
ApoB: Apolipoprotein B-100
CE: Cholesteryl ester
CETP: Cholesterylester transfer protein
FC: Free cholesterol
HL: Hepatic lipase
ITP: Isotransfer point
LPL: Lipoprotein lipase
PL: Phospholipid
